# Pain-related behavior is associated with increased joint innervation, ipsilateral dorsal horn gliosis, and dorsal root ganglia activating transcription factor 3 expression in a rat ankle joint model of osteoarthritis

**DOI:** 10.1097/PR9.0000000000000846

**Published:** 2020-08-25

**Authors:** Valerie Bourassa, Haley Deamond, Noosha Yousefpour, Mary-Ann Fitzcharles, Alfredo Ribeiro-da-Silva

**Affiliations:** a Department of Pharmacology and Therapeutics, McGill University, Montreal, QC, Canada; b Alan Edwards Center for Research on Pain, McGill University, Montreal, QC, Canada; c The Alan Edwards Pain Management Unit, McGill University Health Center, Montreal, QC, Canada; d Division of Rheumatology, Department of Medicine, McGill University Health Center, Montreal, QC, Canada; e Department of Anatomy and Cell Biology, McGill University, Montreal, QC, Canada

**Keywords:** Ankle joint, Monoarthritis, Immunohistochemistry, Imaging, Fiber sprouting, Gliosis

## Abstract

**Introduction:**

Osteoarthritis (OA)-associated pain is often poorly managed, as our understanding of the underlying pain mechanisms remains limited. The known variability from patient to patient in pain control could be a consequence of a neuropathic component in OA.

**Methods:**

We used a rat monoiodoacetate model of the ankle joint to study the time-course of the development of pain-related behavior and pathological changes in the joint, dorsal root ganglia (DRG), and spinal cord, and to investigate drug treatments effects.

**Results:**

Mechanical hypersensitivity and loss of mobility (as assessed by treadmill) were detected from 4 weeks after monoiodoacetate. Cold allodynia was detected from 5 weeks. Using histology and x-ray microtomography, we confirmed significant cartilage and bone degeneration at 5 and 10 weeks. We detected increased nociceptive peptidergic and sympathetic fiber innervation in the subchondral bone and synovium at 5 and 10 weeks. Sympathetic blockade at 5 weeks reduced pain-related behavior. At 5 weeks, we observed, ipsilaterally only, DRG neurons expressing anti-activating transcription factor 3, a neuronal stress marker. In the spinal cord, there was microgliosis at 5 and 10 weeks, and astrocytosis at 10 weeks only. Inhibition of glia at 5 weeks with minocycline and fluorocitrate alleviated mechanical allodynia.

**Conclusion:**

Besides a detailed time-course of pathology in this OA model, we show evidence of contributions of the sympathetic nervous system and dorsal horn glia to pain mechanisms. In addition, late activating transcription factor 3 expression in the DRG that coincides with these changes provides evidence in support of a neuropathic component in OA pain.

## Abstract 2

Supplemental Digital Content is Available in the Text.

In a rat model of osteoarthritis, we found increased joint sensory and sympathetic innervation and glia changes in dorsal horn, accompanying pain-related behavior onset.

## 1. Introduction

Osteoarthritis (OA) is often classified as noninflammatory or degenerative arthritis, in contrast to arthritis with a strong inflammatory component such as rheumatoid arthritis. However, the dichotomy between inflammatory and degenerative arthritis has become unclear as many inflammatory processes have been identified in OA.^60^ Inflammation in OA affects a variety of tissues that are innervated by nociceptors; moreover, cytokines and chemokines have been implicated in the signalling of these nociceptors (for review, see Ref. 48). In addition, overexpression of nerve growth factor causes nociceptive fibers to sprout abnormally into inflamed joints,^47^ whereas anti-nerve growth factor neutralizing antibodies attenuate pain-related behavior.^46^ Studies in inflammatory arthritis show that nerve fibres invade articular cartilage and sprout in the synovial membrane and adjacent bone^23,39,41^ contributing to sensitization. In addition, different OA models have shown similar findings with innervation by Nav1.8-expressing and anti-calcitonin gene-related peptide (CGRP)-positive fibers in the synovium,^51,53^ as well as increased transient receptor potential vanilloid 1 expression in the dorsal root ganglia (DRG).^1^ Although it is likely that innervation changes contribute to the pain in OA, studies in OA models remain limited. Here, in our OA model, we investigated sympathetic and sensory fibre sprouting in subchondral bone and synovium.

In OA patients, there is poor correlation between radiographic changes and reported levels of pain.^14^ Moreover, a subset of OA patients with successful total joint replacement surgeries report sustained levels of pain postrecovery.^42^ Neuropathic pain (NP) is defined as pain after lesion or disease to the somatosensory nervous system (IASP). Indeed, the persistence of pain in some OA patients, after removal of the diseased joint, suggests that this disease can result in neuropathy and central sensitization. In clinical cases of NP, gabapentin is commonly used as a treatment.^68^ Interestingly, gabapentin is analgesic in some OA patients as well, supporting a possible NP-like component.^17^

In animal models, NP is studied by inducing peripheral nerve damage, often of the sciatic nerve.^31^ This consistently results in hypersensitivity that is accompanied by central changes, such as microgliosis, and activating transcription factor 3 (ATF3) expression in the DRG. Microgliosis, for instance, has also been observed in OA models.^37,57^

In this study, we provide an integrated time-course of peripheral and central pathological changes and correlate them with pain-related behavior in an OA model. Those changes include degradation of cartilage, degeneration of bone, sensory and sympathetic fiber innervation changes in the joint, glial activation in the dorsal horn and, at late time point, ATF3 expression in the DRG. To do this, we used the monoiodoacetate (MIA) model in the rat ankle joint, which we characterize for the first time here. The ankle receives most of its nerve supply from the sciatic nerve, which is lesioned in the most commonly used NP models. This facilitates the evaluation of possible mechanisms of OA pain that are also associated with NP. Subsequently, we used pharmacology to suppress components we believe contribute to pain in OA, including sympathetic sprouting, and glial changes.

## 2. Methods

### 2.1. Induction of osteoarthritis

The entire experimental design followed the Care and Use of Experimental Animals of the Canadian Council on Animal Care guidelines. All animals were housed in pairs with soft bedding, food and water ad libitum, on a 12-hour light/dark cycle.

A total of 126 Sprague-Dawley male rats (Charles Rivers Laboratories, Wilmington, MA) weighing 175 to 200 g at the beginning of the study, with 6 animals per group, were anesthetized with 5% isoflurane in O_2_, and given by intra-articular injection in the tibiotalar joint a single dose of 0.8, 1.6, or 2.4 mg of MIA (Sigma‐Aldrich, St. Louis, MO) or saline, in a volume of 40 µL. Doses were selected based on a literature search of MIA concentrations administered in the more commonly used knee joint model of OA.^52^

### 2.2. Behavioral assessments

Pain-related behavior was assessed weekly by a blinded experimenter, whereas treadmill was assessed once each 2 weeks. Before each behavioral experiment, animals were accustomed to the testing environment by being placed in the corresponding cages for at least 30 minutes before testing. The baseline reaction values were measured in the morning of the MIA injections.

### 2.3. Mechanical hypersensitivity

Using a series of calibrated filaments (Stoelting, Wood Dale, IL), the von Frey test was used to assess mechanical hypersensitivity. Animals were placed in individual cages on a mesh wire surface. Filaments were applied using the up/down method^5,15^ on the hind paw both ipsilaterally and contralaterally. The 50% withdrawal thresholds were calculated as previously described.^55^

### 2.4. Cold allodynia

The acetone drop method was used to assess cold pain behavior on the plantar region of the hind paw.^7^ Pain responses were graded as previously described.^18^

### 2.5. Heat hyperalgesia

The Hargreaves apparatus (UGO Basile, Gemonio, Italy) was used to measure latency of paw withdrawal to a heat stimulus, with a cutoff of 20 seconds of heat application, as previously described.^41^

### 2.6. Changes associated with movement

Six animals injected with a dose of 2.4 mg MIA and 6 saline-injected rats underwent a treadmill test (Columbus Instrument Exer 3/6, Columbus, OH) each 2 weeks only (to avoid a training effect^35^). This was performed on a separate cohort of animals to avoid confounds with the other behavior assays. Each rat warmed-up at a speed of 15 m/min with no incline for 10 minutes. The speed was reduced to 10 m/min and increased by 5 m/min every 5 minutes until the rat was unable or unwilling to maintain pace with the treadmill belt. Before week 0, animals received a 5-day daily habituation run with the electric grid as a learning incentive. During experimental days, manual encouragements were used instead (manually touching/flicking the tail).

### 2.7. Histopathology

Rats were perfused intracardially with histological fixatives at 1, 2, 5, and 10 weeks post-MIA for subsequent immunohistochemical processing. Before sectioning, ankle joints were decalcified (see supplementary data for details, available at http://links.lww.com/PR9/A77).

#### 2.7.1. Cartilage degeneration quantification

Cartilage degeneration was visualized in the tibial articular surface of the tibiotalar compartment using the Safranin O (Sigma‐Aldrich) and Fast Green (Fisher Scientific, Waltham, MA) staining method. A hematoxylin and eosin (Sigma‐Aldrich) staining was also used to better visualize chondrocytes. The timeline of cartilage degeneration was quantified by measuring the thickness of cartilage matrix stained by Safranin O with lacunae containing chondrocytes. Using the ImageJ tracing tool, we traced a line over the thickest region of safranin-stained chondrocytes, perpendicular to the surface of the tibia (4 images per animal, n = 6).

#### 2.7.2. Bone degeneration

Ankle joints from the 5- and 10-week post-MIA injection time points were sent to the McGill University Bone Center for microtomography (µCT; using a SkyScan 1072, Bruker Corporation, Billerica, MA) and x-ray imaging (using a Kubtec Xpert 80 apparatus, Stratford, CT), as well as for bone mineral density (BMD; using GE Lunar PIXImus, Madison, WI) measurements (n = 4). Ankle joints were then qualitatively evaluated for signs of degeneration.

#### 2.7.3. Immunohistochemistry and quantification

After decalcification, joints were stained with rabbit anti-CGRP antibody (Sigma‐Aldrich #C8198; 1:2500) or goat anti-vesicular monoamine transporter-2 (VMAT-2) antibody (abm #Y213391, Richmond, BC, Canada; 1:250). Joint sections were incubated in goat anti-rabbit IgG biotinylated antibody (Vector Laboratories #BA-1000, Burlingame, CA; 1:400) and donkey anti-goat IgG biotinylated antibody (Jackson #705-066-147, West Grove, PA; 1:250) for 3,3’-diaminobenzidine immunohistochemistry, and then imaged. CGRP and VMAT-2 fiber innervation densities were measured by a blinded experimenter using the ImageJ software tracing tool. Fibers measuring less than 5 µm long were excluded from analysis to ensure the inclusion of genuine fibers only. See supplementary data for more detailed protocol (available at http://links.lww.com/PR9/A77).

Dorsal root ganglia and lumbar spinal cord were sectioned and stained with rabbit monoclonal anti-ATF3 antibody (Abcam #ab207434, Cambridge, United Kingdom; 1:500), or rabbit anti-ionized calcium binding adaptor molecule 1 (Iba1; Wako # 019-19741, Osaka, Japan; 1:1000) and mouse anti-glial fibrillary acidic protein (GFAP; Cell Signaling # 3670S, Danvers, MA; 1:1000). Dorsal root ganglia and spinal cord sections were subsequently incubated with fluorochrome-labeled secondary antibodies (1:800; Invitrogen, Carlsbad, CA). Dorsal root ganglia slides were incubated with red Nissl (NeuroTrace 530/615 N-21482; 1:100). For the DRG, total number of red Nissl-positive and ATF3-positive cells were counted and averaged for each animal (n = 4). For the spinal cords, extended depth of focus images were analyzed by a blinded experimenter and Iba1+ and GFAP+ cell counts were performed and dimensional ratios (Iba1+ only) were obtained using an ImageJ macro as previously described.^27^ See supplementary data for a detailed protocol (available at http://links.lww.com/PR9/A77).

### 2.8. Pharmacology

#### 2.8.1. Sympathetic block

Vehicle (saline) or 30 mg/kg guanethidine sulfate (Santa Cruz Biotechnology, Dallas, TX) were administered intraperitoneally (i.p.) twice with a 24-hour interval^71^ at 5-week post-MIA injection. The von Frey and acetone tests described above were administered weekly up to 5-week post-MIA injection (predrug) and 4 hours after the second dose of guanethidine. Animals were divided into the following groups: sham + vehicle, sham + guanethidine, MIA + vehicle, and MIA + guanethidine.

#### 2.8.2. Glial inhibitors

Vehicle (10 µL saline), minocycline (100 µg in 10 µL saline; Sigma-Aldrich #13614-98-7, St. Louis, MO), or fluorocitrate (1.0 nmol in 10 µL 2M HCl in saline; Sigma-Aldrich #100929-81-5, St. Louis, MO) were administered intrathecally (i.t.) in the lumbar region of the spinal cord twice with a 24-hour interval at 5-week post-MIA injection. Von Frey and acetone tests (describe above) were administered weekly up to 5-week post-MIA injection (predrug) and 4 hours after the second dose. Animals were divided in the following groups: MIA + fluoro, MIA + mino, and MIA + veh. A second blinded experimenter performed the behavior assessments.

### 2.9. Statistical analysis

All behavioral measurements were analyzed using repeated-measures two-way analysis of variance. Time-courses of changes in innervation, microgliosis, astrocytosis, and microglia morphology were assessed using two-way analysis of variance. Singular comparisons were analyzed with a one-tailed Student *t*-test. For multiple comparisons tests, we used the Bonferroni correction.

## 3. Results

### 3.1. Pain-related behavior changes and establishment of the effective monoiodoacetate dose

**Figure 1.**
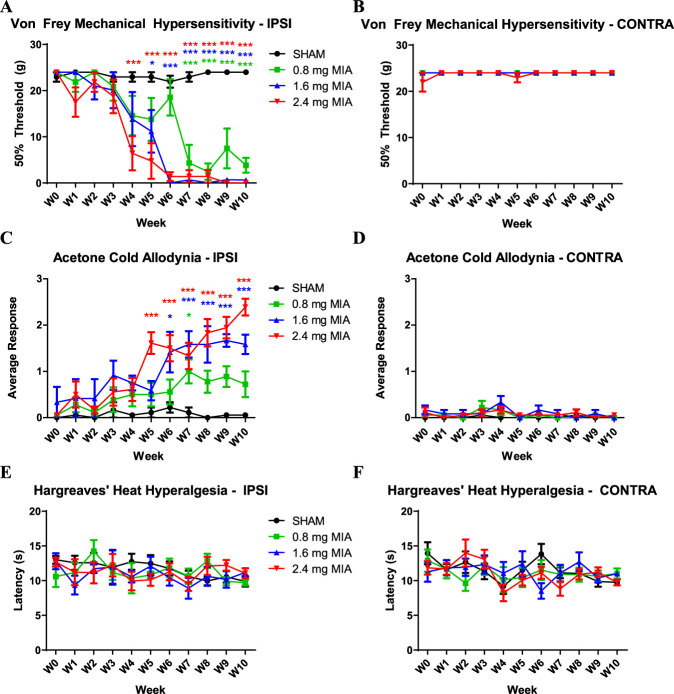
Pain-related behavior in sham rats and animals injected with 3 different doses of MIA using von Frey (A, B), acetone (C, D), and Hargreaves tests (E, F). Mechanical hypersensitivity as tested by the von Frey fibers was first detected at 4-week postinjection using the 2.4 mg MIA dose (*P* < 0.001), while appearing later at 5 and 7 weeks in the 1.6 (*P* < 0.05) and 0.8 mg MIA (*P* < 0.001) groups, respectively (A). The acetone test yielded similar findings, with cold allodynia appearing at the earliest at 5 weeks post 2.4 mg MIA (*P* < 0.001) and at 6 weeks post 1.6 mg MIA (*P* < 0.05) (C). Pain thresholds of the 2.4 mg MIA dose were significantly lower than for the 1.6 mg dose at the 5-week time point (*P* < 0.05). No heat hyperalgesia, as tested by Hargreaves' test, was detected in the ipsilateral paw (E). For all tests, no contralateral effects were observed (B, D, F). Data from (A–F) were analyzed with two-way repeated-measures ANOVA with Bonferroni correction (N = 6). ANOVA, analysis of variance; MIA, monoiodoacetate.

Twenty four Sprague-Dawley rats that were saline-injected, or received either 0.8, 1.6, or 2.4 mg of MIA were tested weekly for 10 weeks to establish dose of MIA for subsequent experiments (Fig. 1A–F). Compared to saline-injected controls, mechanical hypersensitivity became significant at 7, 5, or 4 weeks, when a dose of 0.8, 1.6, or 2.4 mg of MIA was used, respectively (Fig. 1A). It should be noted that with doses of 1.6 and 2.4 mg MIA, from 6 weeks onwards, mechanical hypersensitivity was at the maximum (Fig. 1A). Cold allodynia, assessed with acetone, was consistently detected only with 1.6 and 2.4 mg MIA doses, appearing at 4 and 5 weeks, respectively (Fig. 1C). No heat hyperalgesia was detected by the Hargreaves test in the ipsilateral paw at any time point with any dose (Fig. 1E). In addition, none of the behavior tests revealed contralateral effects (Fig. 1B, D, F). The 2.4 mg dose of MIA resulted in consistent cartilage degeneration across animals (data not shown). Based on this observation and the behavioral outcomes, the 2.4 mg MIA was selected for all subsequent analyses.

### 3.2. Joint function deterioration

**Figure 2.**
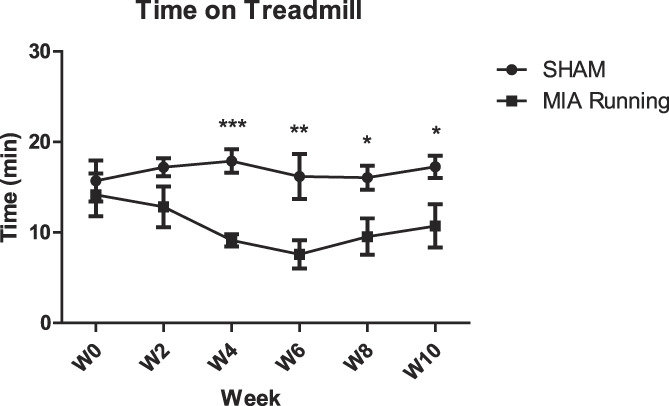
Biweekly running in a treadmill showed that animals treated with 2.4 mg MIA had significantly reduced ability to stay on the treadmill as compared to controls starting at 4-week postinjection (*P* < 0.0001) and lasting until at least 10 weeks (*P* = 0.0175). Two-way ANOVA with Bonferroni correction for multiple comparisons. N = 6. ANOVA, analysis of variance; MIA, monoiodoacetate.

We observed that 2.4 mg MIA-injected animals spent significantly less time on the treadmill starting at 4-week postinjection compared to controls (Fig. 2).

### 3.3. Histopathological changes

**Figure 3.**
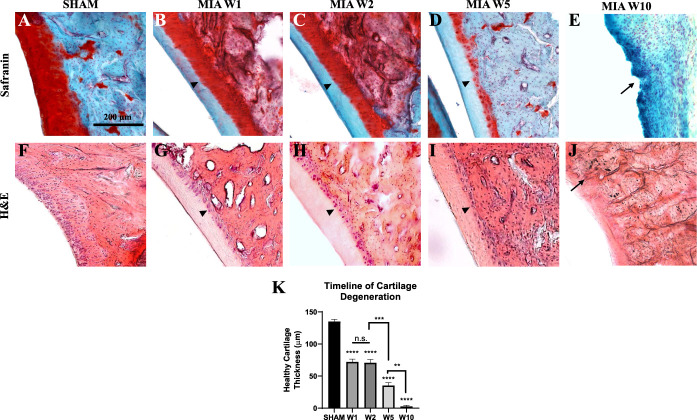
Histopathological changes at 1, 2, 5, and 10 week post 2.4 mg MIA injection, using Safranin O (A–E) and H&E (F–J) stains and quantification of cartilage thinning (K). (A, F) An intact surface of healthy cartilage of SHAM animals. (B–D, G–I) Progressive loss of matrix staining of the outer 2/3 of the cartilage at 1-, 2-, and 5-week post-injection (arrowheads). (E and J) A complete loss of cartilage at 10-week postinjection as only subchondral bone is visible (arrow). Note the disappearance of cartilage and disrupted integrity of subchondral bone (arrows) at 10-week postinjection not detectable at earlier time points. (K) Measurements of intact cartilage thickness over time using the Safranin O stain. As significant difference was observed between SHAM groups of each time point, they were grouped for this figure. There was a strong decrease in cartilage thickness at 1-week post-MIA compared to SHAM controls. No significant difference was observed between week 1 and week 2. Another significant decrease in cartilage thickness was detected at 5-week post-MIA, followed by a complete delamination of cartilage at 10-week post-MIA. Bars represent mean ± SEM; *P < 0.05, **P < 0.01, ***P < 0.001, ****P < 0.0001. Two-way ANOVA with Bonferroni correction for multiple comparisons N = 6. ANOVA, analysis of variance; MIA, monoiodoacetate.

The time-course of histopathological changes was assessed using a Safranin (cartilage) and Fast Green (contrast) staining (Fig. 3A–E) and hematoxylin and eosin staining (Fig. 3F–J). Joints from the control group maintained an intact articular surface, with perfect cartilage integrity, as shown by the intense red Safranin staining (Fig. 3A). Monoiodoacetate-injected animals displayed progressive loss of cartilage matrix staining, as well as considerable cartilage thinning (Fig. 3B–D). Complete loss of the articular cartilage was observed by week 10, leaving the subchondral bone exposed (Fig. 3E). These observations were confirmed by quantifying healthy cartilage thickness over time. A decrease in intact cartilage thickness is observed between SHAM and the 1 week post-MIA time point. There was no statistical difference in cartilage thickness between weeks 1 and 2. However, at week 5, we observed a significant decrease in intact cartilage thickness, followed by total delamination at week 10 (Fig. 3K). The observations with hematoxylin and eosin staining (Fig. 3F–J) confirmed the timeline of cartilage cell death. Damage to the bone in the subarticular cartilage location (subchondral bone), such as disrupted surface integrity, was prominent at week 10, when possible signs of remodelling were seen (Fig. 3E, J).

### 3.4. Radiological and bone mineral density changes

**Figure 4.**
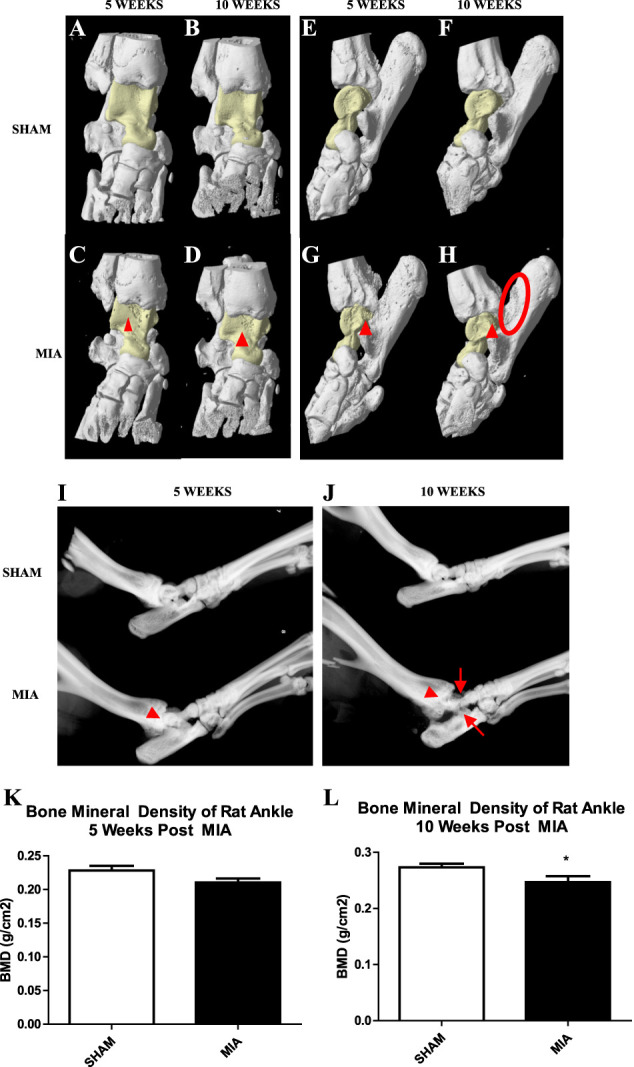
Radiographic changes of the MIA rat ankle joint model of OA. (A–H) X-ray imaging of the rat ankle joints at 5- and 10-week post-MIA injection. (A–H) Microtomography (μCT) of SHAM (A, B, E, F) and MIA-treated joints (C, D, G, H) with the talus bone highlighted in yellow. (A–D) The dorsal view and (E–H) the medial view of the ankle joints. (G, H) Irregular new bony growths on the calcaneus bone (red circle), and fibrillations on the surface of the talus (arrowhead). (C, D) Ventral view of the ankle also shows surface fibrillations in the talus (arrowhead). (I, J) Conventional X-ray images of a SHAM and MIA-treated joint at 5- and 10-week postinjection. The MIA joint at 10-week postinjection shows significant irregularities surrounding the talus and calcaneus bones (arrows). Important loss of joint space is also visible at both time points (arrowheads). (K, L) One-tailed Student *t*-test revealed a reduction in bone mineral density of the ankle joint at 10-week postinjection in MIA as compared to control animals (*P* = 0.0374). No differences were detected at the 5-week time point. N = 4. MIA, monoiodoacetate.

We performed microtomography (μCT) x-ray scans with 3D visualization throughout the entire ankle joint at 5- and 10-week postinjection (Fig. 4A–J). Significant signs of bone erosion and fibrillation (arrows) were observed on the talus (yellow) of MIA animals at 5- and 10-week postinjection (Fig. 4C, D, G, H). Signs of bony outgrowths, indicating osteophyte formation, on the calcaneus bone adjacent to the ankle joint (red circle on Fig. 4H) were visible in the MIA group at 10-week postinjection. Conventional x-ray images (Fig. 4I, J) revealed joint space narrowing in MIA-injected animals as compared to sham animals at 5 (Fig. 4I) and 10 weeks (Fig. 4J).

Bone mineral density scans were obtained (Fig. 4K, L). A small but significant reduction in BMD of the whole ankle joint was detected at 10-week postinjection in MIA compared to control animals (Fig. 4L).

### 3.5. Changes in sensory and sympathetic innervation of joints

**Figure 5.**
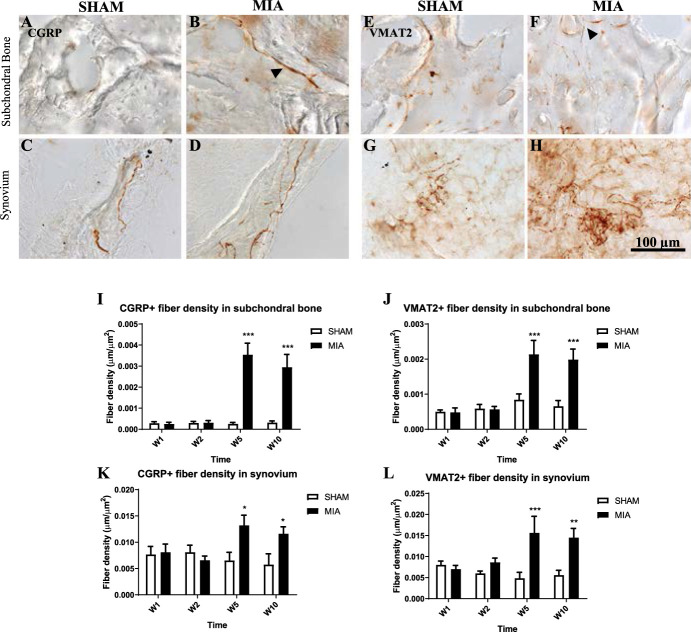
Sensory and innervation as detected by CGRP antibody using bright-field immunohistochemistry in the tibial subchondral bone (A, B) and tibiotalar synovial membrane (C, D) decalcified tissue at 1-, 2-, 5-, and 10-week postinjection of 2.4 mg MIA. (E–H) Sympathetic innervation as detected by vesicular monoamine transporter 2 (VMAT2) using 3,3’-diaminobenzidine immunohistochemistry of the tibial subchondral bone (E, F) and tibiotalar synovial membrane (G, H) decalcified at 10-week postinjection of 2.4 mg MIA. (B, F) Anomalous fibers sprouting outside of haversian canals (arrowheads). Quantification of fiber densities revealed significant increase in innervation of CGRP+ fibers (*P* < 0.001) (I) and VMAT-2+ fibers (*P* < 0.001) (J) in the subchondral bone at 5- and 10-week post-MIA. In the synovium, we observed increases in innervation in CGRP + fibers (*P* < 0.05) (K), and in VMAT-2+ fibers (*P* < 0.001 at 5 weeks, *P* < 0.005 at 10 weeks) (L) at 5- and 10-week post-MIA injection. No significant changes were observed at 1- and 2-week post-MIA. Data were analyzed with two-way ANOVA with Bonferroni correction. N = 6. ANOVA, analysis of variance; CGRP, calcitonin gene-related peptide; MIA, monoiodoacetate.

Peptidergic nociceptive fibre as well in sympathetic fibre innervation was assessed in the tibial subchondral bone of the tibiotalar junction (and in synovial membrane connecting the tibia and talus (Fig. 5A–H). We used 3,3’-diaminobenzidine-based immunohistochemistry with antibodies against the peptidergic nociceptive fiber marker CGRP (Fig. 5A–D) and sympathetic fiber marker VMAT-2 (Fig. 5E–H). Figure 5A–F shows representative images of innervation patterns at 10-week post-MIA or sham injection. We observed that in the OA-associated subchondral bone, VMAT-2 and CGRP nerve fibers sprouted outside the haversian canals (arrows; Fig. 5B, F). Our quantification revealed major innervation increases in the subchondral bone and synovial membrane by both sensory and sympathetic fibers at 5 weeks and 10 weeks (Fig. 5G–J). The increase of innervation was particularly marked for the CGRP-immunopositive fibres in the subchondral bone at 5- and 10-week post-MIA (Fig. 5I). No innervation changes were found at 1- and 2-week post-MIA injection.

**Figure 6.**
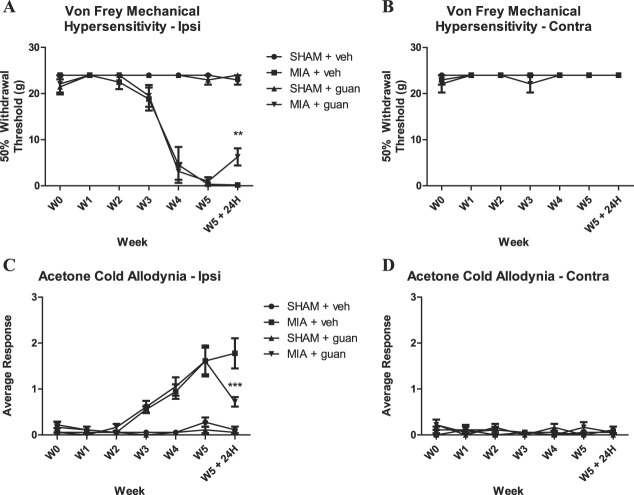
Behavioral assessments after sympathectomy using guanethidine using von Frey (A, B) and acetone (C, D) tests. Note partial alleviation of mechanical hypersensitivity (*P* < 0.005) (von Frey hairs) (A) and cold allodynia (*P* < 0.001) (C) in MIA animals at the 5-week time point, 24 hours after the second injection of guanethidine i.p. Contralateral behavior data are shown in (B) and (D). Data were analyzed with two-way repeated-measures ANOVA with Bonferroni correction. Bars represent mean ± SEM. N = 6. ANOVA, analysis of variance; MIA, monoiodoacetate.

To further investigate the contribution of the sympathetic nervous system to pain-related behavior, we administered guanethidine (known to produce a long-term sympathetic blockade^34,45^), at 5-week post-MIA, a time point where we observed consistent and irreversible pain behavior (Fig. 6A–D). We observed a significant alleviation of both mechanical hypersensitivity and cold allodynia after guanethidine administration (Fig. 6A, C).

### 3.6. Activating transcription factor 3 expression in the ipsilateral dorsal root ganglia

**Figure 7.**
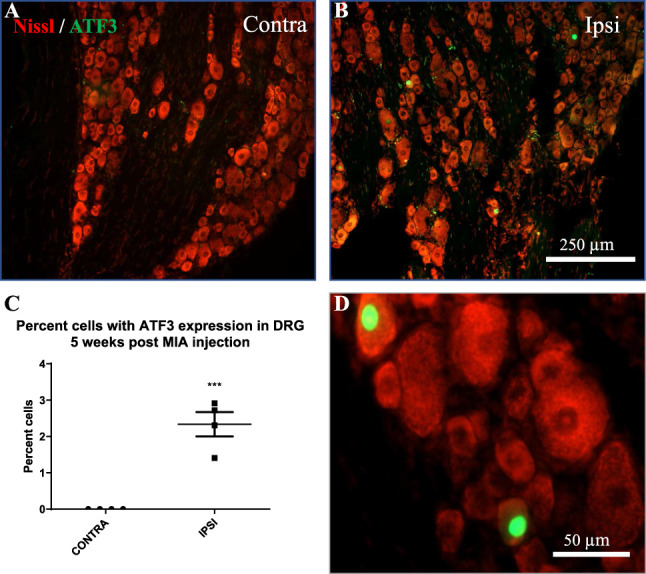
Activating transcription factor 3 (ATF3) expression in lumbar DRG 5-week post-MIA injection. Low-magnification images show no expression of ATF3 in the contralateral DRG (A), and increased expression in the ipsilateral DRG (B), quantified in (C). (D) A higher-magnification image clearly showing localization of the ATF3 signal in the nucleus. Data were analyzed using a one-tailed unpaired *t* test. Bars represent mean ± SEM; ***P < 0.001. N = 4. DRG, dorsal root ganglia; MIA, monoiodoacetate.

To assess neuronal stress associated with the nociception in this model of OA, ATF3 expression in the DRG was investigated (Fig. 7A–C). At 5-week post-MIA injection, the contralateral DRG had no expression of ATF3 (Fig. 7A, C), as in sham animals (data not shown). Interestingly, in the ipsilateral DRG, we observed ATF3 expression in a small, but consistent, number of DRG neurons (Fig. 7B, C).

### 3.7. Changes in microgliosis and astrogliosis in the spinal cord

**Figure 8.**
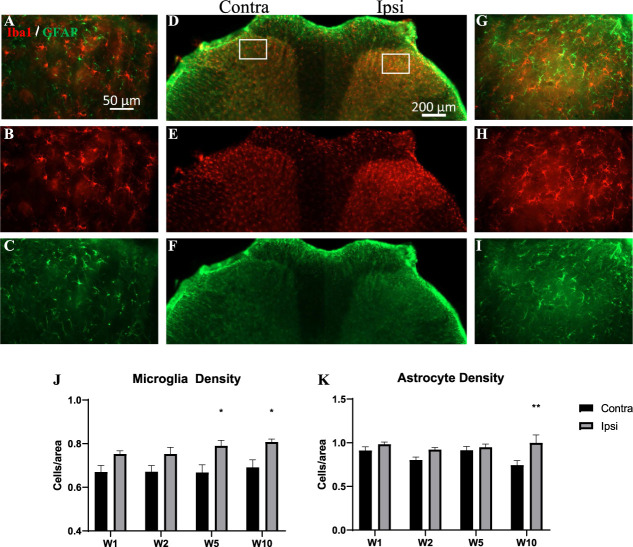
Microgliosis (Iba1) and astrocytosis (GFAP) in the laminae II to III of the spinal dorsal horn. (A–I) Representative fluorescence micrographs showing increased immunoreactivity of Iba1 and GFAP in ipsilateral dorsal horn of MIA-treated animals 10-week postinjection compared to contralateral. (G, E, F) Low-magnification micrographs of the dorsal horn showing where images in (A–C) and (G–I) were obtained (framed areas). (J, K) A time-course of microglia and astrocyte cell densities at 1-, 2-, 5-, and 10-week postinjection. Data were analyzed with two-way ANOVA with Bonferroni correction. Bars represent mean ± SEM; *P < 0.05, **P < 0.01, ***P < 0.001. N = 6. Note also the difference in morphology in the Iba1 immunoreactivity in the ipsilateral dorsal horn compared to the contralateral. ANOVA, analysis of variance; GFAP, glial fibrillary acidic protein; Iba1, ionized calcium binding adaptor molecule 1; MIA, monoiodoacetate.

Microgliosis has previously been detected in the MIA model of OA,^37,57,61^ and in a model of destabilization of the medial meniscus.^65^ The time-course of microgliosis and astrocytosis, in this ankle MIA model, was investigated using Iba1 and GFAP immunoreactivities (Fig. 8A–K). We observed increased microglia cell counts at 5- and 10-week post-MIA injection (Fig. 8J), and an astroglia density increase at week 10 (Fig. 8K). These increases were observed in laminae II to III of the 2 medial thirds of the spinal dorsal horn (Fig. 8A–I), where most primary afferents innervating the ankle joint terminate. As we expected for this OA model, the localization of the gliosis in the dorsal horn matches observations in NP models of the sciatic nerve.^32^

**Figure 9.**
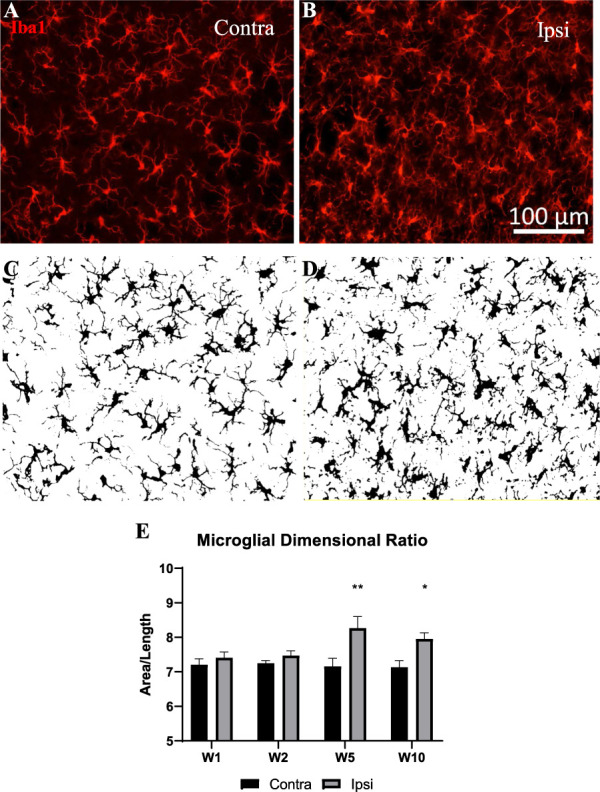
Microglia shape changes. Microglia morphology was assessed by Iba1 immunoreactivity in contralateral (A) and ipsilateral (B) laminae II to III (shown images from lamina II at the 10-week time point). Binary images (C, D) were skeletonized and the dimensional ratio (length of cells/area of cells) calculated by the software. (E) Increased dimensional ratio in the ipsilateral dorsal horn of MIA-treated animals at 5- and 10-week postinjection. Data were analyzed using two-way ANOVA with Bonferroni correction. Bars represent mean ± SEM; *P < 0.05, **P < 0.01, ***P < 0.001. N = 6. ANOVA, analysis of variance; Iba1, ionized calcium binding adaptor molecule 1; MIA, monoiodoacetate.

In addition, we also observed changes in microglia morphology similar to that previously observed by our group in an inflammatory model,^40^ indicative of a reactive microglial phenotype. Indeed, microglia in the ipsilateral dorsal horn had a larger cell body, with retracted processes, compared to mostly ramified cells in the contralateral dorsal horn (Fig. 9A–D). These changes, as assessed by an increased dimensional ratio, were significant at 5- and 10-week post-MIA (Fig. 9E).

**Figure 10.**
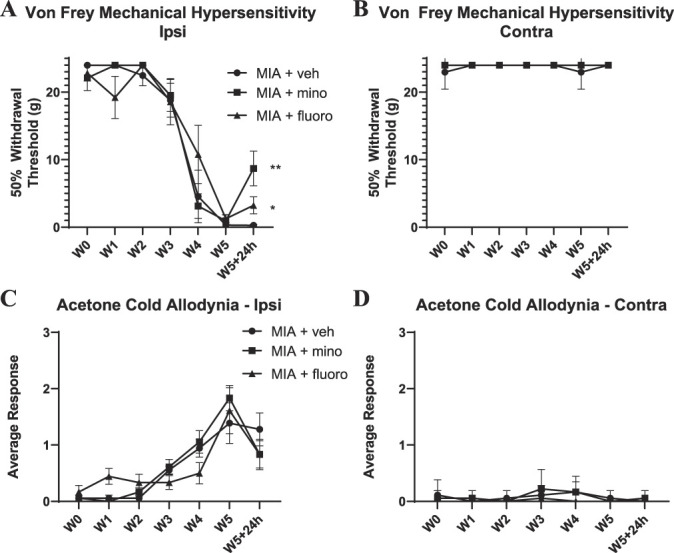
Behavioral assessments after glial inhibitors minocycline and fluorocitrate using von Frey (A, B) and acetone (C, D) tests. Note partial alleviation of mechanical hypersensitivity (A) by minocycline (*P* < 0.01) and fluorocitrate (*P* < 0.05) at the 5-week time point, 24 hours after the second injection of the glial inhibitor. No significant alleviation of cold allodynia was observed (C). (B, D) No contralateral effect. Data were analyzed using two-way repeated-measures ANOVA with Bonferroni correction. Bars represent mean ± SEM. N = 6. ANOVA, analysis of variance.

To understand the contribution of microgliosis and astrocytosis to pain-related behavior, we administered the glial inhibitors minocycline and fluorocitrate at 5-week post-MIA (Fig. 10A–D). Minocycline is widely accepted as a glial inhibitor, proposed to act by preventing the transition of microglia into the proinflammatory state.^36^ However, the effect of minocycline on astrocyte function is unclear. Fluorocitrate is a metabolic toxin that is selectively taken up by astrocytes.^16^ We observed a partial but significant alleviation of mechanical allodynia after both minocycline and fluorocitrate administration, with a larger effect observed with the minocycline treatment (Fig. 10A). No drug effect was observed on the cold allodynia behavioral assessment (Fig. 10C).

## 4. Discussion

We provide an integrated time-course of the development of pain-related behavior and pathological changes in the joint and spinal cord of this OA model of the rat ankle joint. We described the time-course of nociceptive sensory and sympathetic innervation changes and found significant innervation increases at time points in which there was marked mechanical and cold allodynia. Pain-related behavior was also associated with ATF3 expression in the DRG as well as microglia and astrocyte changes in the dorsal horn. Using pharmacology, we suppressed the function of putative contributors to pain in OA, including sympathetic fibers, microglia, and astrocytes. The changes described above include the presence of primary afferent stress and central changes—known descriptors in NP.

### 4.1. Characterization of the ankle joint monoiodoacetate model

In the literature, most rodent models of OA involve the knee joint, given the high prevalence of knee OA in the clinic. With our ankle joint model, direct comparisons to changes in the spinal cord in established NP models can be made because similar nerves are affected. The ankle joint is mostly supplied by branches from the sciatic nerve (tibial, sural, deep peroneal) and saphenous nerves,^21^ with innervation from the spinal cord at the level of lower lumbar regions (L3–L6). The knee joint is supplied by branches from the femoral, tibial, common peroneal, and obturator nerves,^20^ which have different distributions at the spinal cord (L2–L4). Our laboratory has been using this OA model as well as an inflammatory arthritis model of the ankle joint to investigate NP-like features of arthritis in the spinal cord. In fact, the localization of the gliosis observed in this model matched observations in several NP models involving injury to the sciatic nerve.^32^ In addition, we were able to detect cold allodynia, which is seldom reported in knee joint models, although it is clinically relevant.^49,70^ Moreover, loss of mobility was detected, which aligns with clinical investigations that show a strong correlation between pain and functional limitations of the joint in patients with OA.^25,50,69^

### 4.2. Morphological changes in the joint and presence of fiber sprouting

We observed signs of bone remodelling, presence of osteophytes, and joint-space narrowing in this model, which are important changes associated with OA in humans.^2^ Although progressive cartilage loss, bone deformation, and remodelling often result in joint pain and increased functional disability, 50% of patients with these structural changes are asymptomatic.^26^ Conversely, a minority of patients with minimal and even radiographically undetectable damage suffer from OA-related incapacitating pain.^14,33^ One possible explanation for this discrepancy is the existence of a variable neuropathic component to osteoarthritic pain.

We observed increased densities of sensory and sympathetic fibers in the synovium and subchondral bone correlating with pain-related behavior. Persistence of pain in patients with OA has been associated with increased peptidergic free nerve endings in synovial tissue, and high concentrations of the nociceptive peptide, substance P.^58^ We suspect that neurogenic inflammation as well as increased nerve growth factor released by inflammatory cells^44^ and chondrocytes^43^ are major drivers of pain in this model.

The sympathetic sprouting that we observed reproduces abnormal innervation patterns that are observed in neuropathic pain models. In these, we have seen close appositions of sensory and sympathetic fibers in skin of the territory affected by the lesion.^24,73^ Functional studies of sensory-sympathetic coupling show that the sympathetic effect on sensory neurons is excitatory,^4,6,13^ and the abnormal coupling suggests modulation of C-fiber excitability through norepinephrine release.^72^ In fact, sympathetic blockade remains a recommended treatment for some peripheral neuropathies.^28^ In agreement with our previous data using a complete Freund's adjuvant model of the ankle joint,^41^ we observed that sympathectomy partially attenuated pain-related behavior. This change, however, was relatively minor, suggesting that modulation of sensory fibers excitability through the sympathetic system is not sufficient for pain maintenance in this model.

### 4.3. Activating transcription factor 3 expression in the dorsal root ganglia

Activating transcription factor 3 expression in DRG has been described as a marker for neurons axotomized by peripheral nerve injury. Activating transcription factor 3 is not detectable in intact primary sensory neurons,^63,66^ but is consistently upregulated in the nucleus of injured peripheral neurons.^38,66^ In addition to models of peripheral nerve injury, ATF3 has been detected in an in vitro model of diabetic neuropathy,^56^ in a mice rheumatoid arthritis model,^8^ and in a MIA model of OA in the rat knee joint.^30,64^ However, these OA studies only detected ATF3 at early time points, and it is unclear whether this should be interpreted as toxicity from the MIA compound, or axon degeneration caused by the underlying disease. For this reason, we studied ATF3 expression in our MIA model only at 5-week postinjection, a time point where we observed consistent and irreversible mechanical and cold hypersensitivity, as well as sensory fiber sprouting in the periphery. We detected a small but consistent number of ATF3-expressing neurons in the ipsilateral DRG, but not contralaterally. We believe this is a consequence of ongoing OA, which results in hyperinnervation of bone with likely repeated nerve terminal injury in exposed bone. At 5 weeks, it is unlikely that this is a consequence of MIA toxicity, although this could be a factor at earlier time points. Rather, we believe these results indicate the presence of primary afferent injury.

### 4.4. Microgliosis and astrocytosis

Recently, a surge of novel findings attribute an important role to microglia in the development and maintenance of chronic pain.^59^ A common feature of the neuropathic phenotype is disruption of excitatory and inhibitory transmission in the dorsal horn.^11,67^ Activated microglia drive this disinhibition by releasing BDNF, which through its trkB receptor causes the downregulation of the potassium-chloride cotransporter KCC2 in spinal neurons.^10^ In this study, we observed an increased density and activation of microglia starting at 5 weeks. Microglia activity suppression by minocycline partially relieved mechanical allodynia. Together, these findings suggest that proinflammatory changes caused by microglia activation contribute to pain in OA at late stages.

Like microglia, astrocytes enter a reactive state after peripheral nerve injury.^9,22^ However, the participation of astrocytes in MIA models of OA is unclear; some groups report increased GFAP immunoreactivity,^57^ whereas others report lack of response.^54^ In this study, we observed a minor but significant increase in astrocyte number at 10 weeks only, in contrast to 5 weeks for microgliosis. These data align with the finding that activated microglia precede the activation of astrocytes, and may in fact play a role in astrocyte reactivity.^62^ Dimensional ratio of astrocyte morphology was not calculated because GFAP immunoreactivity is not a reliable marker of cell shape. Fluorocitrate, a suppressor of astrocyte activity, partially alleviated mechanical allodynia. A larger effect may have been observed if the drug had been administered at the 10 week time point. Therefore, our data suggest that astrocytes play a minor role in pain in OA.

### 4.5. Limitations

Ankle OA is relatively uncommon compared to knee and hip OA in patients,^12^ which is a limitation of this study. However, we believe the ankle model was the correct model to identify peripheral and central changes associated with the neuropathic-pain phenotype and correlate these changes with pain-related behavior. These findings remain relevant for the clinic: although not very frequent, some patients develop ankle arthritis, predominantly as posttraumatic arthritis.^12,29^ Moreover, the ankle joint model of MIA presented here displays a much slower progression of changes, which allows for a time-dependent analysis of the emergence of features associated with OA. This contrasts with the rat knee joint in which mechanical hypersensitivity has been detected as early as 5 days postinjection with significant cartilage damage at 20 days.^17^ It is important to note that a high number of individuals suffering from OA report pain sensations comparable to those described in neuropathies.^3,19^

## 5. Conclusion

In this study, we investigated the time-course of changes in joint innervation, ATF3 expression in DRG, and spinal dorsal horn glia and found that their onset correlated with pain-related behaviour and extensive structural damage in the joint. We also observed that the pharmacological suppression of sympathetic fiber function, microglia reactivity, and astrocyte function led to mild ameliorations of pain-related behavior. Taken together, our data reinforced the concept that multiple factors are contributing to pain in OA, including features that are commonly described in models of NP. Our objective was to provide a multifacetted time-course of relevant changes in OA using a robust animal model that can be directly compared to animal models of NP. We believe that further investigation of a neuropathic phenotype in OA pain has important implications for the development of therapeutic approaches.

## Disclosures

The authors have no conflicts of interest to declare.

## Appendix A. Supplemental digital content

Supplemental digital content associated with this article can be found online at http://links.lww.com/PR9/A77.
